# Comparisons of Culturally Targeted Food and Beverage Advertisements in Caribbean-American Neighborhood and Non-Latinx White Neighborhood in New York City

**DOI:** 10.1089/heq.2021.0039

**Published:** 2022-02-01

**Authors:** Carla C. Milan, Kirti R. Singh, Angelica Burac, Allison P. Janak, Yuanqi Gu, Marie A. Bragg

**Affiliations:** ^1^ Department of Public Health Nutrition, School of Global Public Health, New York University, New York, New York, USA.; ^2^ Department of Population Health, School of Medicine, New York University, New York, New York, USA.

**Keywords:** health disparities, food/beverage advertisements, targeted advertisements, Caribbean-American

## Abstract

**Purpose:**

This descriptive study aimed to (1) compare the number of food and beverage advertisements (ads) located in a Caribbean-American neighborhood and non-Latinx white neighborhood in New York City (NYC), and (2) qualitatively assess and compare the culturally targeted marketing themes of the food and beverage advertisements in both neighborhoods.

**Methods:**

Three research assistants photographed all outdoor food and beverage advertisements (*n*=361) across a 1.6 kilometer distance on a high-retail street in a Caribbean-American neighborhood and a non-Latinx white neighborhood. We used content analysis to evaluate advertising themes, and sorted food into nutritional categories (e.g., fast food and alcohol). We identified two neighborhoods with similar income levels in Queens, NYC, USA—South Ozone Park residents are predominantly non-white Caribbean Americans based on data from the NYC Department of City Planning, whereas residents of Steinway are predominantly non-Latinx white.

**Results:**

We identified a significantly higher proportion of fast-food advertisements in the Caribbean-American neighborhood (19.78%, *n*=36) compared with the non-Latinx white neighborhood (5.03%, *n*=9; *p*<0.001). Among beverage advertisements, 30.77% (*n*=56) featured alcohol brands in the Caribbean-American neighborhood, whereas 22.91% (*n*=41) featured alcohol brands in the non-Latinx white neighborhood. In the Caribbean-American neighborhood, 24.18% (*n*=44) of food and beverage advertisements referenced Caribbean culture.

**Conclusions:**

The Caribbean-American neighborhood in this study had more fast-food advertisements relative to non-Latinx white neighborhoods. More research is needed to understand the effects of culturally targeted ads on Caribbean-American communities.

## Introduction

Ethnic and racial minorities and immigrant communities experience higher rates of chronic diseases and their corresponding risk factors than non-Latinx white populations. In the United States, for example, cardiovascular disease (CVD) remains the leading cause of death, and accounts for the majority of deaths among American Indian, Asian American, black, and Hispanic men.^1^ Risk factors for CVD include hypertension, obesity, poor diet, smoking, and alcohol consumption, rates of which are high among some immigrant communities.^2–4^ Among immigrants living in the United States who were born in Mexico, Central American, or the Caribbean, 70% meet overweight or obesity criteria.^2^

This represents the highest proportion among all immigrant groups, seven percentage points higher than U.S.-born adults who meet overweight or obesity criteria (63%).^2^ And whereas immigrant communities—such as Caribbean-American populations—often have better CVD outcomes when compared with their U.S.-born counterparts,^2^ this advantage lessens as the length of time spent in the United States increases.^5^ The food environment represents a major contributor to dietary risk factors given the widespread availability and promotions of energy-dense nutrient-poor products.^6–8^

Racially targeted advertisements represent one element of the food environment that shapes dietary behaviors among communities of color. On the one hand, targeted marketing can benefit the targeted group if the advertisements promote products that benefit one's health (e.g., infant car seats). On the other hand, these advertisements may exacerbate health disparities when companies target unhealthy products to communities experiencing high rates of health conditions linked to the consumption of those products.^9–11^ In the case of malt liquor and menthol cigarettes, companies heavily targeted black consumers in advertisements, and exposure to targeted advertisements was positively associated with consumption of these products among black youth.^6,9–13^

Several studies have quantified the use of racially targeted food marketing among black and Latinx communities,^6,13–22^ Asian Americans communities in New York City (NYC),^23^ but no studies have quantified Caribbean-Americans' exposure to targeted advertising, which is concerning given rising rates of diet-related diseases among this group.^3,7,24^

The number of Caribbean immigrants living in the United States has increased over the past four decades; in 1980, the total number of Caribbean immigrants amounted to 1,258,000 residents and increased to 4,415,000 immigrants as of 2017, the majority residing in Florida and New York.^25^ Studying this group is of particular importance given diabetes and CVD affect Caribbean immigrants. Nearly 12% of immigrants from Mexico, Central America, and the Caribbean who were surveyed in the National Health Interview Survey reported having diabetes mellitus, the highest rate among all immigrant groups surveyed.^26^

Given Caribbean-American communities represent a growing proportion of racial and ethnic minorities in the United States, we designed this study to compare the prevalence and healthfulness of food advertisements in a Caribbean-American neighborhood versus non-Latinx white neighborhood.

## Methods

Researchers identified a neighborhood in NYC composed of predominantly Caribbean/Caribbean-American residents. For this study, we defined Caribbean-American populations as individuals who identify as being from countries bordering the Caribbean Sea. Because the U.S. Census does not collect data on Caribbean Americans, we were unable to identify census tract data for the exact percentages of the Caribbean/Caribbean-American populations in NYC neighborhoods.

Instead, we used data from the 2013 Newest New Yorkers report and Population FactFinder, two tools developed by the NYC Department of City Planning. The Newest New Yorkers report identifies the most common nations of origin of foreign-born residents within each NYC neighborhood in addition to the proportion of foreign-born residents in the neighborhood's total population.

We used NYC Department of City Planning's aggregation of census tracts called Neighborhood Tabulation Areas to define each neighborhood surveyed.^27^ We identified Richmond Hill/South Ozone Park, Queens, NY (hereafter, “South Ozone Park”) as the neighborhood with the highest proportion of foreign-born residents (58%) who identify as immigrants from Caribbean nations (72%)^28^ South Ozone Park has a large Indo-Caribbean population that has a strong cultural influence in the area.^29^ The neighborhood is home to the largest population of Guyanese immigrants in New York state, which is second only to Chinese foreign-born residents of Queens.^29^ Liberty Avenue is commonly referred to as “Little Guyana.”^28,29^

To select a retail-dense street for collection of photographs of outdoor advertisements, we identified the densest hub of public transportation and retail outlets in South Ozone Park using subway and bus maps and Google Maps data. We also relied on knowledge of three study coauthors who identify as Caribbean or Caribbean American and were raised in those neighborhoods.

**FIG. 1.**
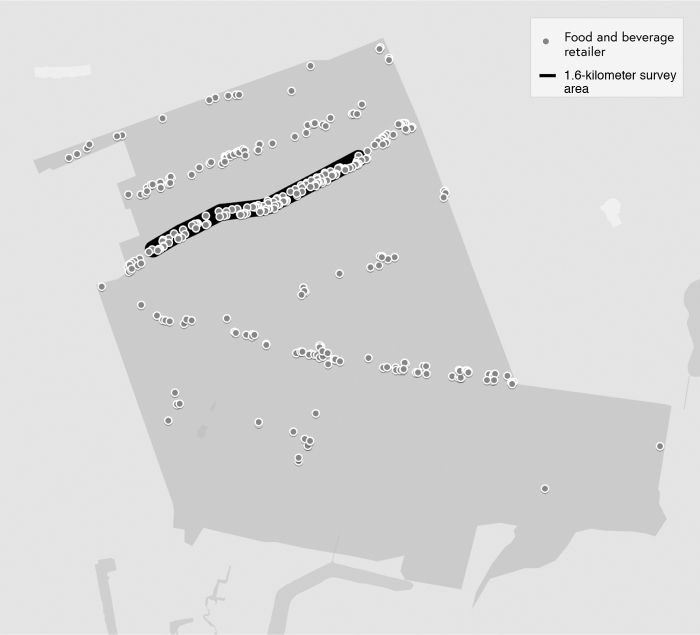
Map of South Ozone Park neighborhood (i.e., Caribbean neighborhood). (Food and beverage retailers included restaurants, delis, fast food chains, convenience stores, bars, specialty food shops, and beer, wine and liquor stores.)

**FIG. 2.**
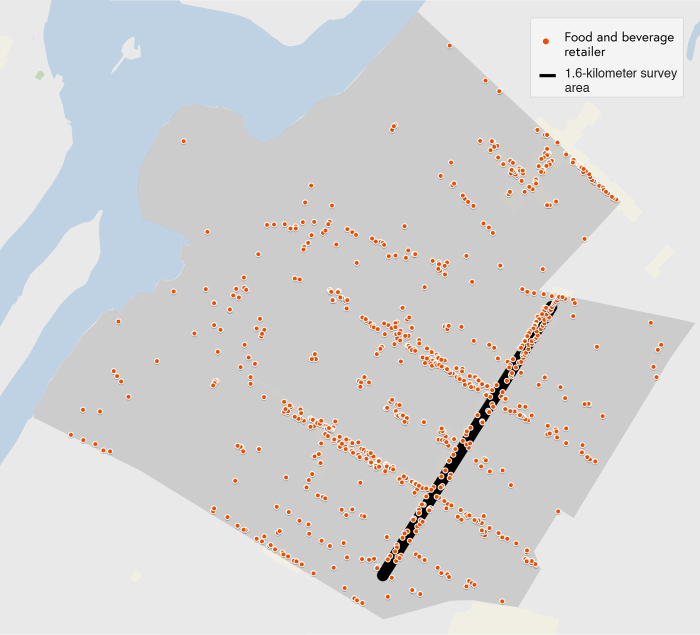
Map of Astoria neighborhood (i.e., Non-Latinx white neighborhood). (Food and beverage retailers included restaurants, delis, fast food chains, convenience stores, bars, specialty food shops, and beer, wine and liquor stores.)

The segments surveyed represented a large portion of commerce present in each respective neighborhood (Figs. 1 and 2). Comparably, each neighborhood had limited retail sites and street advertisements located outside of these sections due to them being primarily residential. Therefore, the selected 1.6 kilometer segments are where residents are likely most exposed to food and beverage advertisements. We selected a 1.6 kilometer segment from 107th Street to 129th Street on Liberty Avenue in South Ozone Park as the first data collection site for the Caribbean-American neighborhood (Fig. 1).

We chose a second data collection site as a control site. We used NYC Department of City Planning's Population FactFinder to find an area with similar household income to that in South Ozone Park, where residents are predominantly non-Latinx white. We selected Astoria, Queens, NY (hereafter, “Astoria”) because 62.8% of the residents are non-Latinx white.^30^ The median and mean household income in Astoria, $63,610 and $82,587, respectively, were similar to the median and mean household income in South Ozone Park, which were $69,406 and $88,535, respectively.^30^

The two neighborhoods also have similar supermarket to bodega ratios (1:8 in Astoria, 1:10 in South Ozone Park), childhood obesity rates (22% in Astoria, 21% in South Ozone Park), and adults reporting their own health as “excellent,” “very good,” or “good” (79% in Astoria, 77% in South Ozone Park).^31,32^ We chose a heavily trafficked retail area of Astoria near public transportation to closely match the selected South Ozone Park location. The location was a straight 1.6 kilometer distance from 25th Avenue to 35th Avenue on Steinway Street in Astoria, Queens, NY (Fig. 2).

Researchers developed a qualitative codebook with six items building upon the methodology of similar research by Bragg et al. that examined the presence of food and beverage advertisements targeting Asian Americans in NYC.^23^ The six codebook items included the type of food/beverage being advertised (e.g., fast food and energy drinks), nutrition claim presence and examples (e.g., fat-free), presence of Caribbean languages (e.g., Spanish and Creole), visual portrayal of Caribbean culture (e.g., island photography), persons featured in the ad (e.g., a famous person), and the presence of a culturally relevant product (e.g., traditional dishes and products from Caribbean nations).

Among a diverse group of 12 coders, there was 100% agreement on the portrayal of Caribbean culture and culturally relevant products. In addition, three coders include the study coauthors who identify as Caribbean American, which informed the coding process.

To classify the nutritional content of products advertised, we sorted food and beverages into categories. The food types were coded by the following categories: cultural product (e.g., Caribbean product), deli/restaurant (e.g., local or cultural restaurant), dessert (e.g., ice cream), fast food (e.g., McDonald's), meat/fish (e.g., raw or halal meat), and produce (e.g., fresh fruit/vegetables). The beverage types were coded by these categories: alcohol, bottled tea, bubble tea, coffee, coconut water, dessert, energy drinks, fast food, juice, soda, and water.

Researchers visited each neighborhood in March 2020 and walked from end-point to end-point on both sides of the street to collect photos of food and beverage advertisements. Advertisements analyzed in this study were displayed on storefront windows, on the sidewalk in front of stores, and on food trucks. There were few billboards in both neighborhoods, none of which displayed food or beverages advertisements. Researchers did not take photos of advertisements located inside stores. Each advertisement was then coded once, unless an identical ad was repeated elsewhere in the field, in which case researchers conferred and applied a uniform coding to all observations of the identical advertisement.

Dessert food and dessert beverage advertisements were distinguished from fast food; dessert food/beverage advertisements from a fast-food establishment were coded only as “dessert” and not coded a second time under the “fast-food” category. This decision was made to isolate dessert food/beverages in the data set, as opposed to broadly categorizing them as “fast-food.” If a single advertisement included images of more than one food and/or beverage item, the advertisement was coded only once as “deli/restaurant” or “fast-food.”

This decision was made to reflect the common nature of advertisements from delis, restaurants, and fast-food establishments. “Fast-food” was defined as a nationally recognized establishment with >15 locations and advertisements coded as such were identified by the presence of their logo (e.g., Burger King, Popeyes, and Papa John's). Data coded as “deli/restaurant,” in contrast, were advertisements for locally owned restaurants that were not fast-food chains.

To assess intercoder reliability, 10.0% of the advertisements were randomly selected and coded by research assistants using the established codebook. Later, researchers ensured the required 90.0% percentage agreement had been met.^33^ Once reliability was established, the remaining data were coded in accordance with the established protocols by the same researchers. The data were analyzed by determining percentages of advertisements in the context of several relevant categories. A chi-square test was conducted to compare the presence of fast food and alcohol ads in the Caribbean-American neighborhood versus the non-Latinx white neighborhood. As this was not human subjects research, we did not require review from the institutional review board at NYUGSOM.

## Results

Researchers photographed a total of 361 advertisements in the two neighborhoods on two retail-dense street segments of 1 mile per neighborhood. The two neighborhoods in which the street segments were located differed in racial composition (63.5% white in non-Latinx white neighborhood vs. 5.2% white in Caribbean neighborhood) and were similar in income level ($73,798 median income level in the non-Latinx white neighborhood vs. $69,028 median income level in the Caribbean neighborhood).

Food advertisements accounted for 60.33% (*n*=108) in the non-Latinx white neighborhood, compared with 48.9% (*n*=89) of advertisements in the Caribbean-American neighborhood (Table 1). Researchers observed a higher prevalence of fast-food advertisements in the Caribbean-American neighborhood (19.78%, *n*=36) than the non-Latinx white neighborhood (5.03%, *n*=9). Cultural food products were more prevalent in the Caribbean-American neighborhood (3.85%, *n*=7), compared with 0.00% (*n*=0) in the non-Latinx white neighborhood. Advertisements representing delis and restaurants accounted for 40.78% (*n*=73) of food advertisements in the non-Latinx white neighborhood, compared with 3.85% (*n*=7) in the Caribbean-American neighborhood.

**Table 1.** Comparison of the Number of Advertisements in Caribbean-American Neighborhood Relative to Non-Latinx White Neighborhood

	Caribbean-American neighborhood					Non-Latinx white neighborhood				
	Total	Nutrition claim		Caribbean cultural appeal		Total	Nutrition claim		Caribbean cultural appeal	
	*n*	*n*	%	*n*	%	*n*	*n*	%	*n*	%
Cultural food/drink	7	3	42.85	5	71.43	0	0	0.00	0	0.00
Food ads (*n*=197)										
Deli/Res^a^	23	0	0.00	12	52.17	73	5	6.85	0	0.00
Dessert^a^	7	0	0.00	3	42.86	21	1	4.76	0	0.00
Fast food^a^	36	0	0.00	0	0.00	9	1	11.11	0	0.00
Meat/fish	9	0	0.00	8	88.89	0	0	0.00	0	0.00
Produce	7	0	0.00	0	0.00	5	0	0.00	0	0.00
*Total food ads*	*89*	*3*	*3.37*	*28*	*31.46*	*108*	*7*	*6.48*	*0*	*0.00*
Beverages ads (*n*=164)										
Alcohol	56	0	0.00	11	19.64	41	0	0.00	0	0.00
Bottled tea	9	1	11.11	0	0.00	0	0	0.00	0	0.00
Bubble tea	0	0	0.00	0	0.00	14	1	7.14	0	0.00
Coffee	1	0	0.00	1	100.00	10	3	30.00	0	0.00
Coconut water	2	1	50.00	2	100.00	0	0	0.00	0	0.00
Deli/Res^a^	3	1	33.33	0	0.00	7	3	42.86	0	0.00
Dessert^a^	3	0	0.00	0	0.00	0	0	0.00	0	0.00
Energy drinks	9	5	55.55	2	22.22	1	2	0.00	0	0.00
Fast food^a^	0	0	0.00	0	0.00	1	0	0.00	0	0.00
Juice	5	2	40.00	0	0.00	2	0	0.00	1	50.00
Soda	3	0	0.00	0	0.00	0	0	0.00	0	0.00
Water	2	0	0.00	0	0.00	2	0	0.00	0	0.00
*Total beverage ads*	*93*	*10*	*10.75*	*16*	*17.20*	*71*	*4*	*5.63*	*1*	*1.41*
*Grand total of food and beverage ads (n=361)*	*182*	*13*	*7.14*	*44*	*24.18*	*179*	*11*	*6.14*	*1*	*0.56*

^a^ The repetition of Deli/Res, dessert, and fast food in the beverage category is defined as the following: dessert beverages: dessert drinks such as milkshakes. Deli/Res beverages: non-dessert homemade beverages from non-fast-food restaurants.

As expected, we identified 16 (17.20%) culturally targeted beverage advertisements in the Caribbean-American neighborhood, and we identified 1 (0.56%) culturally targeted beverage advertisements in the non-Latinx white neighborhood (Table 2). Beverage advertisements accounted for 39.66% (*n*=71) of the total advertisements in the non-Latinx white neighborhood, compared with 51.1% (*n*=93) in the Caribbean-American neighborhood. A higher prevalence of advertisements featuring alcoholic beverages was observed in the Caribbean-American neighborhood (30.77%, *n*=56) in comparison with the non-Latinx white neighborhood (22.91%, *n*=41).

**Table 2.** Breakdown of Caribbean Cultural Appeal Types Present in Food and Beverage Advertisements in Caribbean-American Neighborhood Relative to Non-Latinx White Neighborhood

	Type of Caribbean cultural appeal													
	Caribbean-American neighborhood							Non-Latinx white neighborhood						
	Any Caribbean cultural appeal	Caribbean language^a^		Caribbean imagery^a^		Caribbean product^a^		Any Caribbean cultural appeal	Caribbean language^a^		Caribbean imagery^a^		Caribbean product^a^	
	*n*	*n*	%	*n*	%	*n*	%	*n*	*n*	**%**	*n*	%	*n*	%
Cultural food/drink	5	4	80.00	2	40.00	0	0.00	0	0	0.00	0	0.00	0	0.00
Food ads (*n*=28)														
Deli/Res	12	8	66.67	8	66.67	3	25.00	0	0	0.00	0	0.00	0	0.00
Dessert	3	3	0.00	0	0.00	0	0.00	0	0	0.00	0	0.00	0	0.00
Fast food	0	0	0.00	0	0.00	0	0.00	0	0	0.00	0	0.00	0	0.00
Meat/fish	8	8	100.00	4	50.00	0	0.00	0	0	0.00	0	0.00	0	0.00
Produce	0	0	0.00	0	0.00	0	0.00	0	0	0.00	0	0.00	0	0.00
*Total food ads*	*28*	*24*	*85.71*	*14*	*50.00*	*3*	*10.71*	*0*	*0*	*0.00*	*0*	*0.00*	*0*	*0.00*
Beverage ads (*n*=17)														
Alcohol	11	8	72.73	3	27.27	1	9.10	0	0	0.00	0	0.00	0	0.00
Bottled tea	0	0	0.00	0	0.00	0	0.00	0	0	0.00	0	0.00	0	0.00
Bubble tea	0	0	0.00	0	0.00	0	0.00	0	0	0.00	0	0.00	0	0.00
Coffee	1	1	100.00	1	100.00	0	0.00	0	0	0.00	0	0.00	0	0.00
Coconut water	2	2	100.00	2	100.00	0	0.00	0	0	0.00	0	0.00	0	0.00
Deli/Res	0	0	0.00	0	0.00	0	0.00	0	0	0.00	0	0.00	0	0.00
Dessert	0	0	0.00	0	0.00	0	0.00	0	0	0.00	0	0.00	0	0.00
Energy drinks	2	0	0.00	0	0.00	0	0.00	0	0	0.00	0	0.00	0	0.00
Fast food	0	0	0.00	0	0.00	0	0.00	0	0	0.00	0	0.00	0	0.00
Juice	0	0	0.00	0	0.00	0	0.00	1	1	100.00	0	0.00	0	0.00
Soda	0	0	0.00	0	0.00	0	0.00	0	0	0.00	0	0.00	0	0.00
Water	0	0	0.00	0	0.00	0	0.00	0	0	0.00	0	0.00	0	0.00
*Total beverage ads*	*16*	*11*	*68.75*	*6*	*37.50*	*1*	*6.25*	*1*	*1*	*100.00*	*0*	*0.00*	*0*	*0.00*
*Grand total of food and beverage ads (n=45)*	*44*	*35*	*79.55*	*20*	*45.45*	*4*	*9.09*	*1*	*1*	*100.00*	*0*	*0.00*	*0*	*0.00*

^a^ Caribbean cultural appeal types—language, imagery, and product—present in the advertisements studied are not mutually exclusive. An advertisement may contain more than one type of Caribbean cultural appeal.

Beverages were divided into categories such as dessert, which included milkshakes and other sweetened beverages that differed from juice, soda, bubble tea, energy drinks, and bottled tea. Many of these categories were more prevalent in the Caribbean-American neighborhood, including energy drinks (4.95%, *n*=9), dessert (1.65%, *n*=3), bottled tea (4.95%, *n*=9), juice (2.75%, *n*=5), and soda (1.65%, *n*=3). In the non-Latinx white neighborhood, 9.50% (*n*=14) of beverage advertisements were identified as bubble tea advertisements, compared with 0.00% (*n*=0) in the Caribbean-American neighborhood.

Two categories of advertisements—fast food and alcohol—were more prevalent in the Caribbean neighborhood when compared with the non-Latinx white neighborhood. A chi-square test of independence revealed that the type of advertisements (i.e., fast food, alcohol, or other) and neighborhood demographics (Caribbean American or non-Latinx white) were significantly associated, *χ*^2^ (2)=25.44, *p*<0.001.

**FIG. 3.**
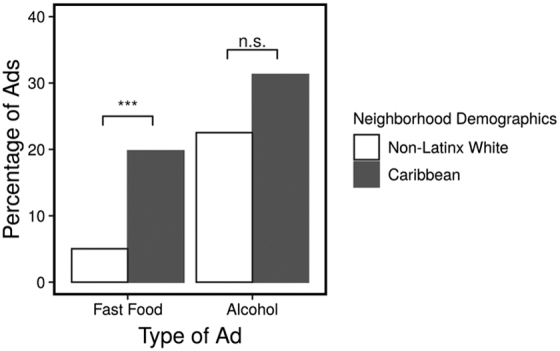
Fast food and alcohol ads by neighborhood demographics.

*Post hoc* analyses based on residuals of the chi-square test showed that there was a significantly higher percentage of fast-food advertisements in the Caribbean neighborhood (19.78%) than in the non-Latinx white neighborhood (5.03%), *p*<0.001, whereas the difference in the percentage of alcohol advertisements was not significant between the two neighborhoods (31.28% in Caribbean American; 22.53% in non-Latinx white), *p*=0.6 (Fig. 3).

Researchers calculated percentages of the prevalence of nutrition claims on advertisements. Nutrition claims were identified by the presence of language suggesting the product featured would improve health, be healthier through the omission of certain ingredients (e.g., sugar, fat, and monosodium glutamate), or contained health-oriented words (e.g., organic). Researchers found that 42.86% (*n*=3) of cultural food products in the Caribbean-American neighborhood made health claims. In the non-Latinx white neighborhood, 6.85% (*n*=5) of deli and restaurant food advertisements contained health claims.

In the Caribbean-American neighborhood, 15.38% (*n*=28) of food advertisements and 8.80% (*n*=16) of beverage advertisements evoked Caribbean culture. Researchers defined three categories to describe these cultural appeals: language, visual, and product. In the total food category of the Caribbean-American neighborhood, the most common cultural appeal device was language, with 13.19% (*n*=24) of all food advertisements using Spanish text of the advertisement. In Caribbean-American neighborhood, 8.80% (*n*=16) of advertisements in the beverage category used a cultural appeal.

**FIG. 4.**
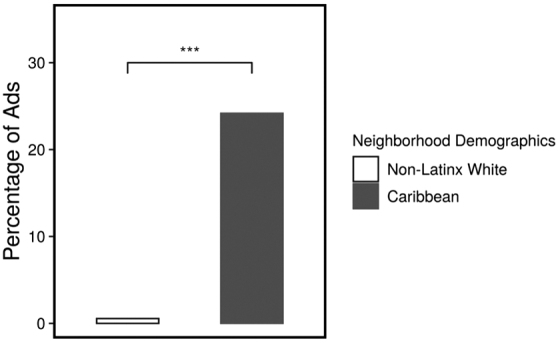
Ads with Caribbean cultural appearance by neighborhood demographics.

Language was the most common cultural appeal device, with 6.04% (*n*=11) of all beverage advertisements using some Spanish text. As expected, there were no Caribbean culturally targeted advertisements observed in the non-Latinx white neighborhood. To examine the association between the dominance of advertisements with Caribbean cultural appearance and neighborhood demographics, a chi-square test of independence was performed. A significantly higher percentage of such advertisements were found in the Caribbean neighborhood (24.18%) than in the non-Latinx white neighborhood (0.55%), *χ*^2^ (1)=43.99, *p*<0.001 (Fig. 4).

## Discussion

Our data revealed that fast-food and alcohol advertisements were more prevalent in the Caribbean-American neighborhood compared with the non-Latinx white neighborhood with similar income levels. Such targeting is concerning because companies promote energy-dense nutrient-poor products more frequently to communities of color than non-Latinx white communities.^6,14,15,17,21–23^

In our sample of a Caribbean-American neighborhood, >40% of food advertisements promoted fast food, making it the largest subset of food advertisements in that neighborhood. Compared with our sample of advertisements in a non-Latinx white neighborhood, the Caribbean-American neighborhood had nearly five times as many fast-food advertisements. This disparity is concerning because exposure to food advertisements for energy-dense nutrient-poor food and beverages is associated with poor diet.^34–37^

In the non-Latinx white neighborhood, the largest subset of food advertisements was deli and restaurant advertisements with 67.59% of food advertisements featuring restaurant products, more than double what was observed in the Caribbean-American neighborhood (25.84%). Fast-food establishments are more likely than delis to sell and market processed and unhealthy foods.^34^ Heavy advertising of fast-food establishments make their products and their affordable prices familiar to consumers, as such, a fast-food advertisement is likely more compelling to a consumer than a deli/restaurant advertisement.^34^

Our data also reveal that there were more advertisements for alcohol in the Caribbean-American neighborhood compared with the non-Latinx white neighborhood. Such targeting of alcohol advertisements aligns with previous research on the increased presence of alcohol advertisements in areas populated by racial and ethnic minorities.^23^

The high density of alcohol advertisements—coupled with the density of other food/beverage advertisements—compounds Caribbean-American exposure to these advertisements, thereby increasing their risk of diet-related illness. In two separate studies, a systematic review of prospective cohort studies and a systematic review of longitudinal study both conducted in 2009, researchers found that exposure to alcohol advertisements were associated with an increased likelihood of drinking among adolescents who did and did not previously drink.^38,39^

We chose to focus on Caribbean-American communities because they are an understudied group in comparison with other racial and ethnic communities. In NYC, for example, Caribbean Americans make up a significant portion of the population, but they do not neatly fall into racial categories that are currently being researched. Individuals from Caribbean nations may not identify as Latinx, particularly in countries with large African and South Asian populations, such as Jamaica or Guyana. Our research emphasizes the importance of collecting data on regionally specific ethnic groups to highlight the impact of health disparities in immigrant communities. More research on Caribbean populations should be done to further explore this issue.

### Limitations

The study is limited to the 1.6-kilometer area of focus for each neighborhood, subsequently limiting the amount of data collected at each location. However, this study benefits from having two locations, allowing for comparative analyses of data to be conducted. Our research highlights the prevalence of targeted advertisements in a neighborhood consisting of predominantly Caribbean Americans, which is a group that is severely underrepresented in public health research. We did not measure any dietary behavior, which could further shed light on the relationship between outdoor advertising, behavior, and health outcomes.

## Conclusions

Policies that reduce outdoor advertisements of energy-dense nutrient poor food and beverage items could be beneficial in higher-risk communities, as they are disproportionately exposed to such advertisements. Although the limitation of advertising is particularly difficult in the United States due to first amendment protections of corporations, investments in improving the food environment to build communities that have access to foods that promote health and are culturally relevant can impact the exposure to advertisements.

Public pressure and creating awareness of the impact of advertising on dietary decisions should be prioritized to appeal to the social responsibility of food companies and the advertising practices they engage in. Outdoor advertisements are also an understudied subject in public health. Bragg et al. concluded that the excessive exposure to outdoor advertisements in an Asian American neighborhood in Manhattan, NYC led to excessive consumption of unhealthy food and beverage products.^23^

Our research emphasizes the importance of collecting data on regionally specific ethnic groups to highlight the impact of health disparities on immigrant communities, and future studies should examine how the dietary habits of Caribbean Americans may be affected by the density of food advertisements in neighborhoods where residents are predominantly Caribbean Americans. Further research should also be conducted to expand upon these findings with a larger sample and a comparison of the food landscape.

## Abbreviations Used

CVD: cardiovascular disease
NYC: New York City
